# Unveiling correlations between aristolochic acids and liver cancer: spatiotemporal heterogeneity phenomenon

**DOI:** 10.1186/s13020-024-01003-y

**Published:** 2024-09-28

**Authors:** Chengxian Li, Xinyu Li, Ming Niu, Dake Xiao, Ye Luo, Yinkang Wang, Zhi-E. Fang, Xiaoyan Zhan, Xu Zhao, Mingxia Fang, Jiabo Wang, Xiaohe Xiao, Zhaofang Bai

**Affiliations:** 1 Department of Liver Disease, Fifth Medical Center of Chinese PLA General Hospital, Beijing, 100039 China; 2 Evidence-Based Medicine Center, Tianjin University of Traditional Chinese Medicine, Tianjin, 301617 China; 3 Haihe Laboratory of Modern Chinese Medicine, Tianjin, 301617 China; 4 School of Pharmacy, Chengdu University of Traditional Chinese Medicine, Chengdu, 610075 China; 5 Department of Hematology, The Fifth Medical Center of Chinese PLA General Hospital, Beijing, 100071 China; 6 School of Traditional Chinese Medicine, Capital Medical University, Beijing, 100069 China; 7 School of Traditional Chinese Medicine, Southern Medical University, Guangzhou, 510515 China; 8 Department of Pharmacy, Chongqing Hospital of Traditional Chinese Medicine, Chongqing, 400021 China; 9 National Key Laboratory of Kidney Diseases, Beijing, 100039 China

**Keywords:** Aristolochic acid, Liver cancer, Spatiotemporal heterogeneity

## Abstract

Aristolochic acids are a class of naturally occurring compounds in Aristolochiaceae that have similar structural skeletons and chemical properties. Exposure to aristolochic acids is a risk factor for severe kidney disease and urinary system cancer. However, the carcinogenicity of aristolochic acids to the liver, which is the main site of aristolochic acid metabolism, is unclear. Although the characteristic fingerprint of aristolochic acid-induced mutations has been detected in the liver and aristolochic acids are known to be hepatotoxic, whether aristolochic acids can directly cause liver cancer is yet to be verified. This review summarizes the findings of long-term carcinogenicity studies of aristolochic acids in experimental animals. We propose that spatiotemporal heterogeneity in the carcinogenicity of these phytochemicals could explain why direct evidence of aristolochic acids causing liver cancer has never been found in adult individuals. We also summarized the reported approaches to mitigate aristolochic acid-induced hepatotoxicity to better address the associated global safety issue and provide directions and recommendations for future investigation.

**Supplementary Information:**

The online version contains supplementary material available at 10.1186/s13020-024-01003-y.

## Introduction

In 1993, a weight-loss clinic in Belgium used herbal medicine containing aristolochic acid for treatment, resulting in severe kidney damage in multiple patients [1]. The incident drew attention to aristolochic acid, which subsequent research identified as a potent carcinogen linked to kidney failure and urothelial carcinoma. Several studies have since investigated aristolochic acid nephropathy (AAN) and aristolochic acid-induced bladder and upper urinary tract cancers [2–6].

In 2017, aristolochic acid was highlighted again when the journal ‘‘Science Translational Medicine’’ featured an article titled ‘‘The Dark Side of Herbal Medicine,’’ which linked aristolochic acid and its derivatives to liver cancer across Taiwan and Asia, establishing a decisive association [7]. This has raised public concern about whether traditional Chinese medicines containing aristolochic acid could induce liver cancer. Currently, 24 types of aristolochiaceae are listed in the Chinese Pharmacopoeia and other standards, including 14 from the *Aristolochia* genus and 10 from the *Asarum* genus (Suppl. Mat. Appendix-1). In the registered traditional Chinese medicines associated with aristolochic acid, 176 products contain *Asarum* (Suppl. Mat. Appendix-2), and 47 products contain *Aristolochia* (Suppl. Mat. Appendix-3). Given the frequent use of Asarum in decoctions and the broad market for these formulations, the safety concerns surrounding aristolochic acid are highly sensitive and have significant implications for public health and the development of traditional Chinese medicine. To date, several studies have established a link between aristolochic acid and liver damage. However, to our knowledge, these studies have not been systematically reviewed. Here, we explore the mechanisms through which AAs cause hepatotoxicity, the link between AAs and liver cancer, and how to reduce AA-related toxicity.

### Uncertain relationship between aristolochic acid and liver cancer

**Fig. 1:**
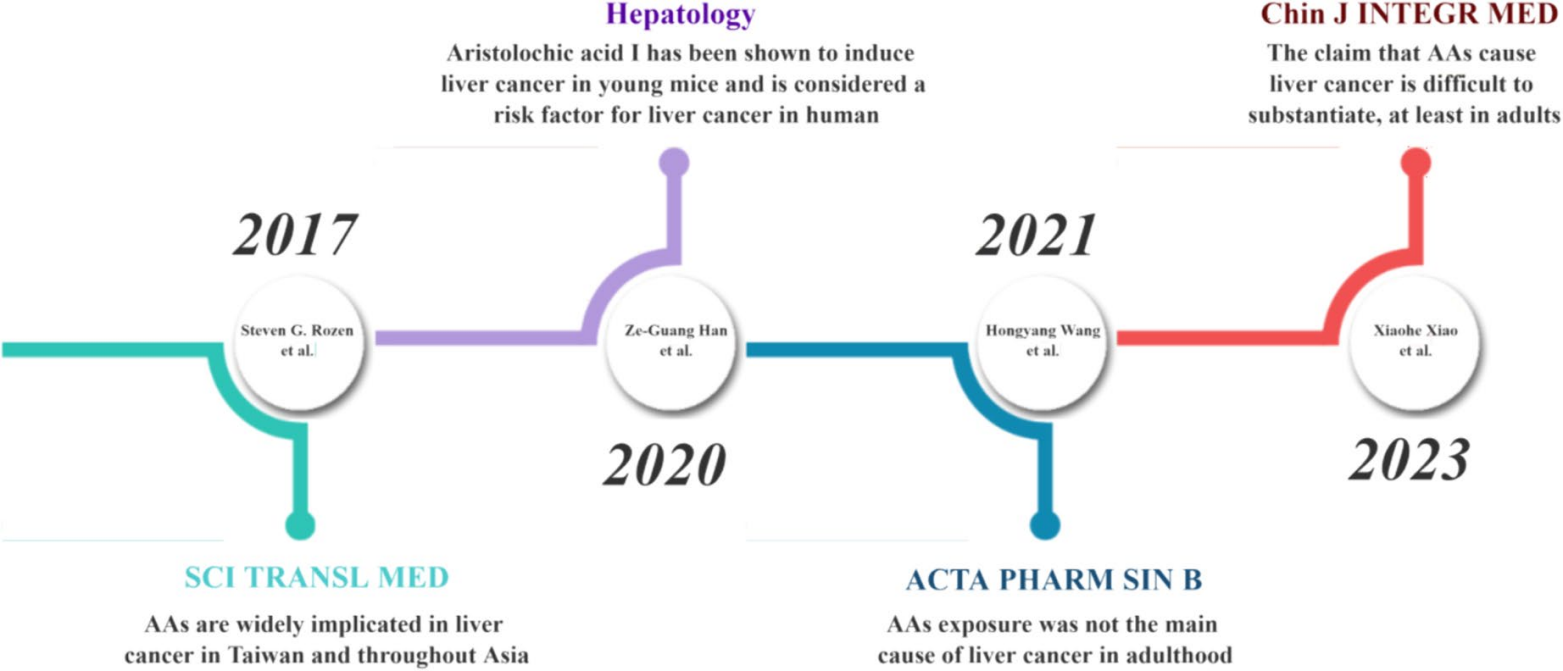
Timeline of major findings relating to whether AAs cause liver cancer

The AA mutation signature characterized by A: T to T:A changes (single-base-substitution mutational signature 22, or SBS22) in hepatic cancer cells was first reported by Poon et al. in 2013 [8]. The study identified 10 patients with SBS22 in the published genome data of 88 Chinese patients with liver cancer, supporting a causative role of AA in liver cancer. Ng et al. [7] described this further in 2017 and argued that poisonous herbs containing AA are important causes of liver cancer in Asia. Their study used exome sequencing technology to detect whole exons of 98 patients with liver cancer in Taiwan. They found that 76 (78%) of 98 patients had characteristic AA-related mutations. They also examined 1400 hepatocellular carcinoma (HCC) samples among publicly available data from China, Japan, Korea, Southeast Asia, North America, and the prevalence of SBS22 varied from 1.7% to 56% of these countries (In Japan, an Asian country, the prevalence of SBS22 is only 2.7% among HCC patients) [7]. Similar results were reported by Letouzét et al. [9] who found an SBS22 detection rate of ≤ 5% in 44 patients from France and in 264 HCC samples from Japan. However, that AA is widely associated with liver cancer in Asia is difficult to conclude based on the above evidence alone. To prove that AA causes liver cancer, two key findings are required: animal experiments showing that AA can directly cause liver cancer, and more rigorous evidence (DNA adducts) of AA exposure in liver cancer patients. Several groups have systematically analysed the results of long-term carcinogenicity studies of AAs in experimental animals to determine a possible connection. Figure 1 shows the timeline and viewpoints of each study.

Han et al. [10] found that aristolochic acid I (AAI) can directly and dose-dependently induce HCC and HCC combined with intrahepatic cholangiocarcinoma (cHCC-ICC) in 14 day-old male C57 mice. Furthermore, DNA adduct detection and shallow whole-genome sequencing of 11 mice with AA-induced HCC and eight with non-neoplastic liver tissues showed that AA can form DNA adducts in young mice and cause the SBS22 fingerprint. To determine whether AA causes liver cancer in humans, the authors further analysed mutation fingerprints of the human tumour genome in various countries to ensure assessment accuracy. Analyses of the genomic data of 1,957 patients with HCC from various countries and regions revealed that Chinese HCC patients are the population most significantly affected by AA. The contribution rate of SBS22 was also generally high among individuals, with it predominating in some patients, suggesting that AA alone causes liver cancer in some individuals. Overall, these results provided direct evidence for AA-induced liver cancer in animals and suggest that AA could be a risk factor for liver cancer in humans. Although the study confirmed that AA can cause liver cancer in experimental animals, it does not directly answer the question of whether AA is the main cause of liver cancer in the Asian population.

A study [11] led by the Wang Hongyang academic team in 2021 provided an answer to this question. The prevalence of 7-deoxyadenosine-N6-yl aristolactam I (dA-ALI, CAS number: 127191-86-0) in liver cancer samples from multiple centers across mainland China was determined to be 5.1%. A further study of AA signature mutations identified SBS22 in 41 of 107 randomly selected patients with HCC. However, only nine of them had tumours with SBS22 predominance among all somatic cell mutations. Wang et al. confirmed that although small doses of AA can mildly damage hepatocytes, they do not cause liver tumours over the long term in adult mice [11]. That is, small doses of AA had no long-term hepatocarcinogenic effects in adult mice, whereas the severity of renal fibrosis worsened with increasing doses of AA. However, AA had hepatocarcinogenic effects in infant mice.

Xiao et al. assessed the link between AA and liver cancer in an objective study [12] of 337 patients with confirmed AAN (male, n = 118; female, n = 219; average age, 55.47 ± 11.01 years) who had a median cumulative estimated AA dose of 1404.0 mg. Follow-up started from 1 year after the administration of herbs and preparations containing AA, and the median was 14 years. No liver tumours were found by the end of follow-up, but 39 of 337 patients with AAN developed tumours. Among these, 34 (87.17%) were urinary system tumours comprising bladder cancer and upper urothelial cell carcinoma, and five were other types (thyroid, lung, bone, and breast cancer, and lymphoma). These results generally agreed with previous findings on the incidence of urinary cancer in populations exposed to AA in the Wenzhou region of China, which are very similar [13]. Repeated checkups revealed that none of the patients with AAN had a confirmed diagnosis of liver cancer during outpatient admission, inpatient treatment, or follow-up after discharge. These findings of a cohort study of AAN confirm that substantiating AAs as a causative factor in liver cancer is difficult at least in adults.

In addition to the studies described above, we have summarized the findings of long-term carcinogenicity studies of aristolochic acid on the liver and kidneys in clinical trials or animal experiments. Table 1 presents these data to facilitate a more intuitive comparison of the research findings.

**Table 1:** Summary of evidence supporting aa-induced liver or kidney cancer in experimental animals and humans

Experimental model	Exposure details or analysis method	Results
Population-based case–control study [4]	Mass spectrometric determination of AL–DNA adducts	dA-AL-I adducts in 76% of Taiwanese ccRCC patients were detected
C57 male mice [10] Age: 14 days	Intraperitoneal injection, multiple doses	AA dose-dependent degrees of liver tumours in all groups
C57 male mice [11] Age: 8 weeks	Gavage every other week for 8 months, multiple doses	Slight hepatocellular damage, but not liver tumour development in the long-term
C57 male mice [11] Age: 14 days	Single gavage AA (10 or 20 mg)	Liver cancer in infant mice
C57 male mice [14, 15] Age: 8 weeks	Daily intraperitoneal injection for 5 days	Severe renal injury; no significant liver injury
C57 male mice [14, 15] Age: 14 days	Daily intraperitoneal injection for 5 days	Varying degrees of cysts found in liver; no significant renal injury
C57 male mice [16] Age: 5–6 weeks	Gavage three times a week for six weeks	Renal cancer was found but no liver cancer
337 patients with AAN [12]	Retrospective analysis of previous exposure to AAs, confirmed AAN	None with AAN had confirmed liver cancer during entire follow-up

*AA* aristolochic acid, *AAN* aristolochic acid nephropathy, *ccRCC* clear cell renal cell carcinoma

### Spatiotemporal heterogeneity in the carcinogenic effects of AA

**Fig. 2:**
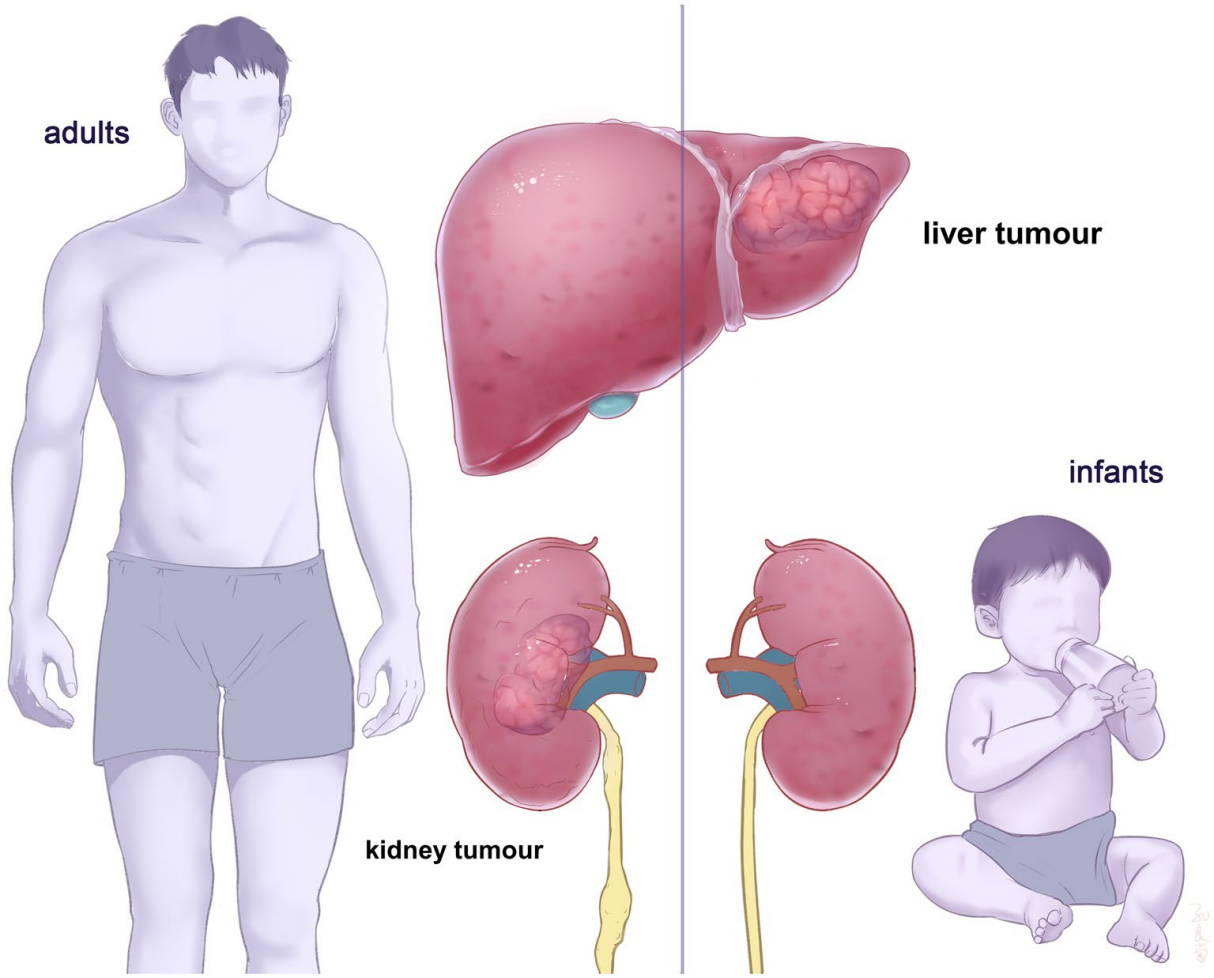
Spatiotemporal heterogeneity of AA carcinogenic effects. Aristolochic acids cause liver, but not kidney tumours in infants, however it cause kindey, but not liver tumours in adults. AA, aristolochic acids

Table 1 shows that AA hepatocarcinogenicity was not evident at adult experimental animal level or humans, whereas a small dose of AA resulted in liver cancer in 14-day-old infant mice. Xiao et al. therefore proposed that the carcinogenic effects of AA are spatiotemporally heterogeneous [14] (Fig. 2). That is, the responses of target organs, such as the liver and kidney to the carcinogenicity of AA at various stages is significantly and spatiotemporally heterogeneous.

Briefly, AA damages the liver in young individuals and kidneys in adults. Therefore, we investigated the characteristics and differences in AA-induced liver and kidney damage in mice of different ages based on the above inferences [15]. The results revealed significant variations in the responses of mice to AA toxicity in the liver and kidney (spatio) at different ages (temporal), which further confirmed the spatiotemporal heterogeneity of the carcinogenic effects of AA. These results have also been confirmed in other scholars' research [4, 16]. This phenomenon explains why no direct evidence of AA exerting hepatocarcinogenic effects has been found in adults, even though a mechanism does exist. AAs induce liver cancer only in infants, and this demographic has not been included among studied clinical patients with HCC to date. Therefore, as we stated in a recent Letter to the Editor: if AA is the primary cause of liver cancer in China and Asia, then children should not be exposed to it [17].

Xiao et al. [14] believe that the phenomenon of spatiotemporal heterogeneity can be mainly explained as follows: the liver, being the main site for drug metabolism and detoxification, has a weak metabolic capacity during infancy, making it more susceptible to injurious responses and leading to damage after exposure to AA or its metabolites. In adulthood, the liver's metabolic function is more established, enabling it to avoid damage by generating an adaptive response. Additionally, the kidney, which plays a crucial role in drug elimination and detoxification, is also an accumulation site for DNA adducts. The immune system, underdeveloped in early life, has weak levels of immune recognition and response to adducts, resulting in minimal renal damage from AA in infancy. However, with a fully developed immune system in adulthood, there is a relatively powerful recognition and response to AA adducts, and a continuous damage response can injure the kidneys. Urothelial tissue, which has a fast turnover rate and rapid regeneration in the event of damage [18], is continuously exposed to potentially carcinogenic metabolites. These conditions may lead the uroepithelium to accumulate somatic mutations through repeated cell renewal, potentially inducing renal, bladder, and/or uroepithelial cancers [19, 20].

Other interesting phenomena have been observed, but whether they contribute to spatiotemporal heterogenei ty remains unknown. Elledge et al. [21] demonstrated that immune escape is also highly tissue-specific. Hence, investigating whether spatiotemporal heterogeneity is associated with specific AA immune escape during different ages and in various tissues is required. Human liver stem cell-derived extracellular vesicles in vivo and in vitro, and human liver stem cell-derived extracellular vesicles (HLSC-EVs) remarkably reduce overexpression of the pro-fibrotic genes α-Sma, Tgfb1 and Collagen I. An investigation using a fibrosis gene array found 35 and 14 profibrotic genes that were respectively upregulated in mice with AA lesions and downregulated after HLSC-EV treatment. Histological findings have shown that such treatment significantly reduces AA-induced tubular necrosis, interstitial fibrosis, CD45 cell infiltration, and fibroblast infiltration [22]. This could also explain the spatiotemporal heterogeneity in the carcinogenic effects of AA. The spatiotemporal heterogeneity mechanism underlying AA-induced liver and kidney damage remains hypothetical and requires further clinical and experimental confirmation. However, the hepatotoxicity and carcinogenic effects of AA in infants have been extensively confirmed. In the following sections, we review the main sources of hepatotoxicity from AA and further discuss its potential carcinogenic mechanisms.

### Potential mechanisms of AA-induced liver cancer

AAs are mixtures of structurally-related nitrophenanthrene carboxylic acids extracted from Aristolochiaceae, which harbour several compounds [23] Table 2 shows that AAs are cytotoxic and genotoxic, and this review analyses laboratory-based key evidence for both types of liver toxicity [10, 24–27]. The bioactivation of AA in the liver relies on various metabolic enzymes. Quinone oxidoreductase (NQO1) is the most potent cytosolic nitroreductase that activates AA in vitro and in vivo. [28] Cytochrome P450 1A1 and 1A2 in human liver microsomes also participate in the metabolic activation processes of AA [29]. Activated AA generates aromatic amine cation intermediate with a non-localized positive charge, which subsequently covalently binds to DNA to form an adduct [30]. The DNA adduct of AAI, 7-(2′-deoxyadenosin-N6-yl)aristolactam I (dA-AL-I) on the non-transcribed chain causes an A:T to T: A transformation characteristic of AA, that is, SBS22. This mutation and reference mutational profiles are described by the Catalogue of Somatic Mutations in Cancer [31]. The hepatotoxic effects and metabolites of AA are well understood; however, the nature of their relationship to the carcinogenic effects of AA remains unclear. This issue continues to be controversial, and in this section, we analyse only the potential mechanisms of AA-induced liver cancer.

**Table 2:** Summary of experimental evidence supporting AA-induced cytotoxicity and genotoxicity

Organism	Exposure details	Findings
SD Rats [24]	AA 2, 4, or 20 mg/kg in DMSO or Blank (DMSO) by gavage for 28 days	Oxidative stress triggered mitochondrial apoptosis with marked tendency toward liver cell infiltration and fibrosis
C57BL6/J [10]	PBS i.p.; multiple doses and dosing times	AA dose-dependently caused hepatic DNA strand breaks within 3 h of administration
C57BL/6 [25]	2 mg/kg in corn oil i.p.; 4 or 8 weeks	AA activated hepatic immune inflammatory system accompanied by immune cell infiltration
Big Blue transgenic rats [26]	Oral gavage doses: 0.1‒10 mg/kg 12 weeks	AA induced mutations and DNA adduct formation in rat liver
HEPG2 [27]	AA: 0–20 μg/mL 24 h DMSO dissolution	AA induced chromosomal aberrations and DNA strand breaks in HEPG2 cells. Subsequent cell arrest in S phase

*AA* aristolochic acid, *DMSO* dimethyl sulfoxide, *HEPG2* hepatoblastoma cell line G2, *i.p.* intraperitoneal injection, *PBS* phosphate-buffered saline

AAs are nephrotoxic and carcinogenic in rodents [32] and cause CHN, now known as AAN in humans. Such neuropathy is characterized by renal interstitial fibrosis and is associated with a high risk of urothelial carcinoma [12, 33]. Some researchers suggest that the carcinogenicity and nephrotoxicity of AA might be independent processes, as evidenced by a case report describing urinary tract cancer associated with AA use, but without apparent renal failure [34]. However, it is not comprehensive to assume that the nephrotoxicity of AA is not involved in the carcinogenic process, as most cases of uroepithelial carcinoma due to AAN are found in patients with end-stage renal disease [35]. Predictably, the carcinogenic process of AA involves both cytotoxicity and genotoxicity. In discussing the mechanism of AA hepatocarcinogenesis, we propose that alongside the genetic mutation perspective, the role of inflammatory cancer transformation should also be considered. For instance, damaged cells can initiate chronic inflammation, which persists if the initial causative factors are not (or cannot be) removed. This inflammation can then extend to adjacent healthy tissues. Over time, such persistent, low-grade inflammation may evolve into fibrosis and eventually lead to hepatocellular carcinogenesis [36].

### Theory of gene mutation

Mutations in somatic genes can cause cancer, but most mutations occurring during DNA replication and cell proliferation in both normal and cancerous cells are functionally neutral, These mutations are not selected for and do not contribute to cancer progression; thus, they are termed passenger mutations. The production and maintenance of the malignant biological tumor-cell phenotype rely on the activation of one or more oncogenes. The genes that facilitate this process are called driver genes, which include proto-oncogenes and tumor suppressor genes. The frequency of mutations in driver genes is much higher than that of random mutations; therefore, they are considered as the root cause of cancer. The dA-AL-I adduct can lead to A:T → T:A reversal genome-wide [2, 8] as well as Tp53 that acts as a tumour suppressor [37, 38]. The A:T to T:A transition is rare in other transitional cell carcinoma mutations listed in the IARC database (http://p53.iarc.fr/), which is why this mutation is considered a characteristic AA mutation [39, 40]. Characteristic mutations of AA have also recently been detected in patients with liver and bladder tumours in Taiwan and other Asian countries, indicating that gene mutations caused by AA are a potential pathway for malignant tumours [5, 7].

### Theory of inflammatory transformation

Chronic HBV infection is a major risk factor for the development and progression of HCC, accounting for > 50% of all HCC worldwide [41, 42]. Tissue repair after injury is complex and metabolically demanding. The various complex and sophisticated tasks that inflammatory cells undertake at sites of injury include wound debridement and the production of chemokines, metabolites, and growth factors [36]. The repair outcome is often imperfect, accompanied by various degrees of fibrosis, [43] which forms due to the abnormal accumulation of collagenous connective tissue. The formation of a fibrotic extracellular matrix disrupts cell polarity and stimulates their proliferation, creating ideal conditions for cancer development [44, 45] Therefore, we speculate that inflammatory-cancer transformation is also involved in AA-induced liver cancer [36, 46–49].

The hepatotoxicity and genotoxicity of AAs are inextricably linked to cellular inflammation, as evidence supports the notion that cell death (necroptosis) triggers or amplifies inflammation [50]. Oxidative stress is a classic pathway of mitochondrial apoptosis [51, 52]. AAs can cause mitochondrial apoptosis via oxidative stress and damage-associated molecular patterns (DAMPs) generated by damaged mitochondria in liver cells; this can significantly amplify the immunological response and release inflammatory cytokines, resulting in chronic liver inflammation [53, 54]. Single-cell sequencing results, enriched by gene sets and regulatory network analyses, suggest that inflammatory responses mediated by signal transducer and activator of transcription 3 (STAT3) and nuclear factor kappa b (NF-κB) signalling might be activated in mouse hepatocytes exposed to AA. In addition, AA can induce infiltration by M1 macrophages and CD8^+^CTL cells in the liver [29]. An increased abundance of these cell types might be related to increased liver inflammation and fibrosis [55–57]. AA triggers DNA damage, and poly(ADP-ribose) polymerase 1 (PARP-1) is mostly associated with a protective function when accumulated DNA damage activates the repair mechanism [58]. It also plays an important role in several aspects of DNA damage, inflammation, and cellular necrosis [59]. AA leads to oxidative stress-associated DNA damage through glutathione (GSH) depletion and extracellular signal-regulated kinase (ERK1/2) pathway activation [60]. Further, it significantly increases the expression of PARP-1 protein in the kidneys of rats [61]. Reactive oxygen species (ROS) initiate DNA single-strand breaks, and PARP-1 is subsequently activated to synthesize the nuclear enzyme poly(ADP-ribose) synthetase (PARS), that leads to poly(ADP-ribosyl)ation, ultimately leading to extreme energy depletion and necrotic cell death. Extracellularly released PARS might simultaneously excite macrophages, causing them to produce cytokines and chemokines in mice and humans [62, 63]. Thus, PARS released by damaged cells might act as a DAMP, which immune cells perceive as a danger signal that leads to an inflammatory response. Inflammation will generate additional ROS, forming a loop to further maintain the inflammatory response, eventually leading to carcinogenesis.

### Collaboration of gene mutations and inflammatory transformation

**Fig. 3:**
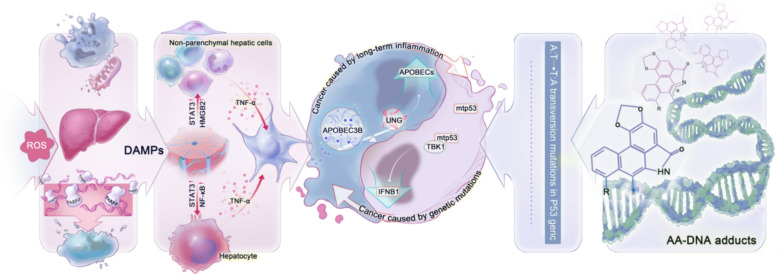
Sources and mechanisms of AA carcinogenicity. Mechanism of AA-induced liver cancer through (left) inflammatory-cancer transformation and (right) gene mutation. Centre, dynamics of these interrelated mechanisms

Gene mutations and inflammatory transformation are key drivers of liver cancer, intricately linked to AAI-induced liver cancer. Figure 3 shows the mechanism of hepatocarcinogenesis and the principle of interconversion between them. The effects of tumour-suppressor gene mutations in cancer cells are not limited to proliferation. Mutated p53 proteins not only lose their ability to suppress tumours but also reduce the degree of infiltration by anti-tumour response mediators (CD8^+^ and natural killer [NK] cells) and promote macrophages towards M2 type polarization that changes the tumour immune microenvironment [64]. M2 macrophages play an important role in the transition from chronic inflammation to fibrosis in AAI-induced inflammatory transformation [56, 65].

Inflammatory cancer transformation is accompanied by gene mutations. Sia et al. found that almost 25% of 956 patients with HCC expressed inflammatory response markers [66] Furthermore, when the inflammation-induced mutation-promoting gene apolipoprotein B mRNA editing enzyme catalytic subunit 3B (APOBEC3B) and the repair gene uracil N-glycosylase (UNG) are imbalanced, the risk of hepatitis progression to liver cancer increases [67]. Although whether AAI causes liver cancer remains unknown, the activated inflammatory signalling pathway stimulates the overexpression of APEBOCs during HBV inflammatory transformation, which causes numerous HCC-related somatic mutations [68, 69]. Therefore, the notion that AA causes liver cancer as a result of genetic mutation combined with inflammatory cancer transformation is reasonable in an era where cancer is fully linked to immunity. The two are interrelated and not separate and distinct. Thus, AA-induced liver cancer has a scientific basis. However, due to the spatiotemporal heterogeneity in the carcinogenic effects of AA, direct evidence has never been found in adults in large-scale cohort studies. Therefore, we conclude that AA (or rather, using herbal medicines containing AA) is not the main cause of liver cancer in China.

### Approaches and potential protective mechanisms against AA hepatotoxicity

The toxicity of AA is not a new topic; it is characterized as being highly carcinogenic, and adducts that are formed persist in the renal tissues of patients and remain detectable decades after AA exposure [70]. Therefore, the best way to deal with this highly carcinogenic substance is to prohibit contact with it [71]. The European Medicines Evaluation Agency, United States Food and Drug Administration, and other agencies have issued warnings against drugs containing AA, [72] and have blocked the import and sale of raw materials and finished products known or suspected to contain it. Nevertheless, despite government warnings, products containing AA persist in the market. Even though reducing exposure to AA is important, some countries such as China, Japan, and South Korea have a long history of using Chinese herbal medicines containing AA and have assigned special value to them [73] Reducing AA toxicity under such circumstances is particularly important. The demand for products containing AA and their perceived pharmaceutical value is such that detoxification of these products has been proposed to allow for their safe use [74].

The latest version of the Chinese Pharmacopoeia 2020 has deleted traditional herbal medicines containing AA, except *Asarum*, which is commonly used in clinical practice in China [75]. Therefore, the potential risks of *Asarum* (especially the parts with medicinal applications) require urgent clarification and new methods to detoxify this product must be found. Microfluidic microarray technology has shown that the hepatic biotransformation of AA leads to ~ fivefold increase in toxicity in human and rat proximal tubular epithelial cells compared with its direct contact [76]. Therefore, to reconsider potential protective measures and mechanisms is particularly important from the perspective of AA liver toxicity. Thus, we propose the concept of interaction of components, targets, and effects to reduce the toxicity of Chinese medicines containing AA (Table 3) [77].

**Table 3:** Summary of main agents and their protective mechanisms against AA toxicity

Direction	Mechanisms of action	Agent	Findings
Component interaction	Electrostatic attraction and π − π stacking	Berberine [78]	Virtually eliminates AA acute nephrotoxicity
Target interaction	Induce NQO1 expression	Dicoumarol [79, 80]	Reduces AA toxicity
	Inhibit CYP1A1/CYP1A2 expression	β-Naphthoflavone [81, 82] Baicalin [81]	Reduces AA-induce liver and kidney damage
	Inhibit AA uptake	Probenecid [84] Wogonin [85] Wedelolactone [85]	Improves renal injury in AA model mice
Effect interaction	Inhibits ROS production and reduces inflammatory damage	L-FABP [86] Resveratrol [87] Ursolic acid [87]	Reduces oxidative stress that protects mice from AA-induced nephrotoxicity Reduces AA-induced nephrotoxicity in zebrafish

*AA* aristolochic acid, *LFBP* liver-type fatty acid-binding protein

### Attenuation of AA toxicity based on component interaction

Component interactions among combinations of herbs regulate the dissolution and transformation of toxic substances. Chinese herbal medicines containing berberine (BER) neutralize the toxic effects of herbal medicines containing AA. Further systemic toxicology studies of mice and zebrafish have revealed that the supramolecular formation of BER and AA self-assembly can considerably lessen AA toxicity and prevent acute kidney impairment [78]. This can be studied more extensively by screening Asarum along with popular clinically compatible medicinal materials, and AA reduction can be detected by liquid chromatography-mass spectrometry. Reducing AA toxicity at the source requires guidance based on the experience and rules of traditional Chinese medicine.

### Attenuation of AA toxicity based on target interaction

**Fig. 4:**
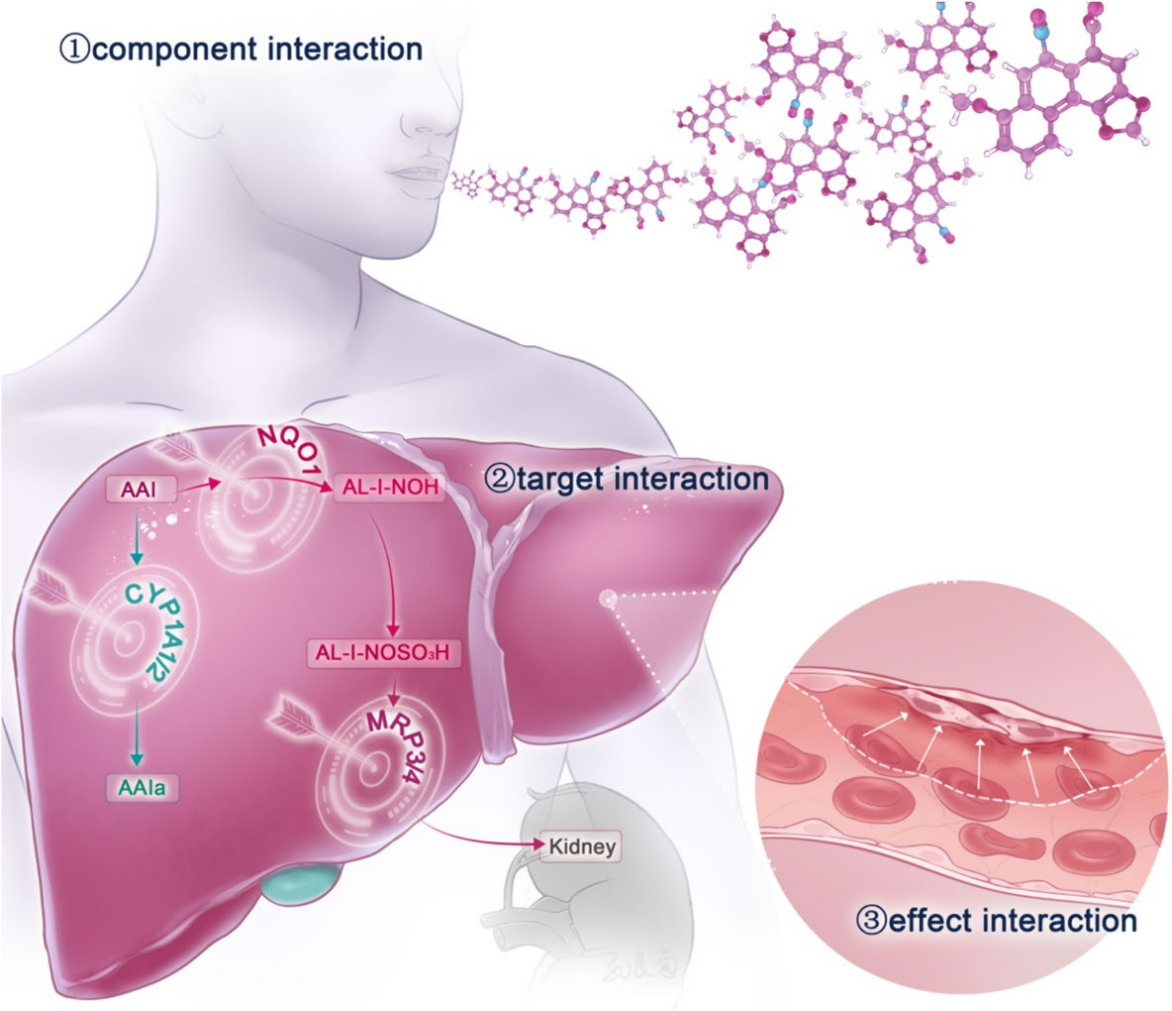
Potential protective approaches against AA based on component, target, and effect interactions. Component interaction: decoct herbal combinations to reduce leaching of toxic substances. Target interaction: reduce AA toxicity by regulating proteins in its metabolic and transport pathways. Effect interaction: antagonize AA-induced inflammatory reactions to attenuate toxicity

The metabolic and transport pathways of AA have reached consensus [76]. The toxicity of AA can be reduced by regulating AA-related metabolic enzymes and transporters (Fig. 4). This has been confirmed in numerous studies in vivo and in vitro. Quinone oxidoreductase is involved in reductive AA activation, which leads to increased toxicity. Dicoumarin inhibits NQO1, and can reduce AA-DNA adducts catalysed by NQO1 by ~ 99%, [79] AA-induced injury in hepatocytes co-cultured with coumarin (10 μM) by ~ 39%, [76] and renal injury caused by AA in vitro [80].

CYP1A1/2 is involved in the oxidation of AA, which directs AA metabolism towards the detoxification direction. The aryl hydrocarbon receptor (AHR) agonist β-naphthoflavone stimulates CYP1A1 expression [81]. Administering C57BL/6 mice with β-naphthoflavones increases the expression of hepatic and renal CYP1A1 or hepatic CYP1A2, which decreases AA toxicity [82]. The natural flavonoid baicalin derived from *Scutellaria baicalensis* also induces CYP1A1/2. It has been observed that after pre-treatment of C57BL/6 mice with baicalin (80 or 160 mg/kg), the expression of CYP1A1/2 in the liver increased, thus attenuating AA nephrotoxicity [83].

Transporters are involved in the movement of AA metabolites. Multidrug resistance proteins (MRPs) 3 and 4 are responsible for excreting metabolites out of the liver, and organic anion transporters (OATs) 1 and 2 are responsible for importing a combination of metabolites and albumin into the kidney. Probenecid is an organic anion transporter 1 and 3 inhibitor that also inhibits the entry of AA through OAT1/2, reduces the formation of specific AA-DNA adducts, and attenuates AA-induced plasma creatinine elevation and intertubular qualitative damage in vitro [84]. Wogonin and wedelide in natural medicines are potent OAT inhibitors that mitigate kidney injury in AA mouse models [85]. Our literature search found no investigative screens for MRP3/4 inhibitors in natural medicines.

### Attenuation of AA toxicity based on effect interaction

The action of AA on the human liver results in several effects including the production of ROS and immune inflammation. Therefore, drugs with anti-oxidative and anti-inflammatory effects can reduce damage caused by AA at the effect level. Liver-type fatty acid-binding protein has an endogenous antioxidant function. Human liver-type fatty acid-binding protein (HL-FABP) transgenic mice treated with AA have lower levels of N(ε)-(hexanoyl)lysine (considered a lipoxygen stress marker at the start of oxidative stress) and heme oxidase-1 production, as well as impaired renal function compared with wild-type mice. These findings indicated that decreasing oxidative stress allows L-FABP to shield mice against AA-induced nephrotoxicity. [86]. Resveratrol and ursolic acid are natural compounds with antioxidant and anti-inflammatory properties, respectively, that decrease AA-induced nephrotoxicity in zebrafish by downregulating the expression of the pro-inflammatory genes and myeloperoxidase, limiting intrarenal blood cell formation, raising glomerular filtration rates, and reducing glomerular sclerosis [87].

## Discussion

In China, interpreting the correlation between Aristolochic acid and liver cancer is particularly complex. This complexity arises because liver cancer often progresses from chronic liver diseases, and there are 78 million hepatitis B virus carriers in China [88]. The role and research status of Aristolochic acids (AAs) in these individuals is worth investigating. Wang et al. took into account HBV-related factors in the animal model and showed that AA did not additively affect the development of liver tumours in C57BL/6-TgHBV [11]. A further real-world study found that in a cohort of 127 patients with, and 9850 patients without HCC in Yinzhou District, located in Ningbo of Zhejiang Province, HBV was an important risk factor for liver cancer, not AA. Similarly, an obvious irrelevancy was found between the consumption of *Asari *Radix (A traditional Chinese herbal medicine containing trace amounts of aristolochic acid) and HCC development both in patients with and in those without HBV infection by Fang et al [89]. However, another retrospective study, adjusted for hazard ratios, showed a significant dose–response relationship between the intake of aristolochic acid and hepatocellular carcinoma (HCC) in patients with HBV infection, suggesting that AA may play a driving role in liver cancer caused by HBV [90]. Based on this cohort, Chen et al. also investigated the role of AA in liver cirrhosis, finding that HBV-infected individuals consuming herbal products containing AA had a higher risk of developing liver cirrhosis, indicating that AA exposure may increase the risk of liver cirrhosis [91]. Furthermore, research by Wang et al. indicated that exposure to aristolochic acid I is associated with poor prognosis in liver cancer patients [92]. Overall, the role of AAs in the progression of chronic liver diseases and liver cancer primarily focuses on clinical and epidemiological studies, and it is difficult to ascertain from existing literature whether AAs contribute to this process. Future research should intensify in this area to explore whether aristolochic acid plays a role in the development of liver cancer through liver cirrhosis and hepatitis B virus infection.

The carcinogenic effects of AAI exhibit complexity and diversity across temporal and spatial dimensions, involving variations in genes, phenotypes, and microenvironments of different target organs at various ages. Understanding these intricate mechanisms across time and space continues to pose significant challenges for researchers. As spatial genomics technology advances, it paves the way for understanding the regulation of genomic elements and the relationships between gene expression, cell function, and cell fate determination [93, 94].

Using spatial genomics technology, scientists have explored the mechanisms by which AAI causes liver and kidney damage. Wang et al. were the first to use spatial metabolomics to confirm that aristolochic acid primarily damages the renal cortex in mice [95]. Furthermore, Chen et al. combined spatial transcriptomics and spatial metabolomics to precisely pinpoint the regions and metabolic changes associated with AAI-induced kidney damage. They found an increased proportion and colocalization of damaged proximal tubules and immune cells (T lymphocytes and macrophages) in the cortex region. During AAI-induced kidney damage, purine metabolism played a crucial role in metabolic reprogramming and served as a potential biomarker for the onset and progression of AAN [96]. Guo et al. used spatial metabolomics to discover that AAI affects taurine and hypotaurine metabolism, glycerophospholipid metabolism, D-glutamine and D-glutamate metabolism, and arachidonic acid metabolism pathways in the mouse liver [97].

Based on existing research, we have found that studies on spatial proteomics of AAI predominantly focus on the kidneys, with damage precisely localized to the proximal tubules. However, the specific sites of AAI-induced damage in the liver remain unidentified. Each region of the liver also possesses distinct functions, one of the best known examples of liver metabolic processes carried out by zonated enzymes is ammonia detoxification [98]. Therefore, investigations into the spatiotemporal heterogeneity mechanisms of AAI carcinogenicity should initially focus on the liver and kidney damage sites. This approach will help narrow the scope of the research. Additionally, in China, aristolochic acid is commonly ingested as an herbal remedy. However, there are differences in the toxicological response to AAI between diseased and healthy states in humans. Consequently, data derived from healthy animals may not adequately predict the drug's risk levels in patients [99]. To address this, we introduce the concept of Disease-Syndrome-Based Toxicology, which utilizes real-world clinical and "pseudo-clinical" disease models as evaluation platforms. This approach allows us to compare the drug toxicity sensitivity and tolerance across different physiological state models, thereby scientifically assessing and predicting the safety of traditional Chinese medicine [100]. To explore the mechanisms of AAI's spatiotemporal heterogeneity, we propose a feasible pathway: selecting traditional Chinese medicine models that include AAI-containing herbs, and focusing on the primary areas of liver and kidney damage through spatial proteomics. By delving into the genetic, proteomic, and metabolic layers, we aim to uncover the mysterious variations of aristolochic acid-induced liver and kidney damage. This will enhance our scientific understanding of aristolochic acid toxicity and aid in developing risk management strategies.

The safety of herbs or plants containing aristolochic acid is not only a concern in China but also a current global issue. Approximately 6% of maize and wheat flour samples from Serbian and Bulgarian endemic regions have tested positive for AA. [101] Analytical methods such as liquid and gas chromatography have detected AA in crops, soil, water, air, and other settings [102–104]. AA can also migrate from *Aristolochia* to soil and be further adsorbed onto other (edible) plants [105, 106]. Considering the widespread prevalence of environmental AA, further investigation is needed to minimize AA-induced liver and kidney damage. Several compounds and natural medicines described herein have hepatoprotective effects. These are of special significance for countries such as China, Japan, and Korea with a long history of applying herbal medicines containing AA and might improve its safe clinical use. However, AAN is usually diagnosed during routine clinical examinations of people who are passively exposed to AA, and no effective preventive or treatment measures are available. In addition, most of the mitigation measures described herein have been applied to experimental animal models of acute rather than chronic injury due to AA exposure.

## Conclusion

Based on available evidence, the mechanism of AA-induced hepatotoxicity is not fully understood, although hepatic damage caused by AA has been investigated in vitro and in vivo*.* We conclude that AA has hepatocarcinogenic potential, but is not a major cause of liver cancer in China, and the carcinogenic effects of aristolochic acid on the liver and kidneys exhibit spatiotemporal heterogeneity. This phenomenon explains why direct evidence of its hepatic toxicity has never been found in adult individuals. Thus, the mechanisms underlying spatiotemporal heterogeneity require further investigation and the true impact of AA exposure on the incidence of liver cancer worldwide requires exploration.

## Supplementary Information

Supplementary material 1

## Abbreviations

AAs: Aristolochic acids
AAI: Aristolochic acid I
AAN: Aristolochic acid nephropathy
DAMPs: Damage-associated molecular patterns
HBV: Hepatitis B virus
HCC: Hepatocellular carcinoma
cHCC-ICC: HCC combined with intrahepatic cholangiocarcinoma
PARP-1: Poly(ADP-ribose) polymerase 1
PARS: Poly(ADP-ribose) synthetase
SBS22: Single-base-substitution mutational signature 22
