# Supplementary material for: Unveiling correlations between aristolochic acids and liver cancer: spatiotemporal heterogeneity phenomenon
Source: Chin Med. 2024 Sep 28;19:132. doi: 10.1186/s13020-024-01003-y (PMC11439320; doi:10.1186/s13020-024-01003-y)
Supplement: Supplementary file 1 — Supplementary material 1 [file 13020_2024_1003_MOESM1_ESM.docx]

**Supplementary Material**

**Appendix-1**

**List of 24 *Aristolochiaceae* medicinal materials**

| 序号 | 药材名 | 原植物基源 |
| --- | --- | --- |
| 1 | 大叶青木香 | 大叶马兜铃A.austrozechuanica |
| 2 | 大百解 | 土木香A.chuii |
| 3 | 朱砂莲 | 四川朱砂莲A.cinnabarina |
|  |  | 朱砂莲A.tuberosa |
| 4 | 九月生(朱砂莲) | 广西朱砂莲A.Tuberosa C.F.Liang et S.M.Hwang |
| 5 | 天仙藤  (马兜铃藤) | 北马兜铃A.contorta |
|  |  | 马兜铃A.debilis |
| 6 | 马兜铃 | 北马兜铃A.contorta |
|  |  | 马兜铃A.debilis |
| 7 | 防己 | 异叶马兜铃A.heterophy1la |
|  |  | 川南马兜铃A.austrozechuanica |
|  |  | 穆坪马兜铃A.moupinensis |
| 8 | 汉防已 | 异叶马兜铃A.heterophy1la |
| 9 | 淮通 | 穆坪马兜铃A.moupinensis |
|  |  | 木香马兜铃A.moupinensis |
| 10 | 木防已  (水城木防已) | 木香马兜铃A.moupinensis |
|  |  | 卵叶马兜铃A.ovatifatia |
| 11 | 木香马兜铃 | 木香马兜铃A.moupinensis |
|  |  | 穆坪马兜铃A.moupinensis |
|  |  | 木香马兜铃A.moupinensis |
|  |  | 藏马兜铃(藏木通)A.griffithii Yhoms ex Duchartre |

| 序号 | 药材名 | **原植物基源** |
| --- | --- | --- |
| 12 | 大青木香 | 广西马兜铃A.kwangsiensis Chun et How |
| 13 | 冕宁防己 | 穆坪马兜铃A.moupinensis Franch |
| 14 | 寻骨风 | 绵毛马兜铃A.mollissima |
| 15 | 苕叶细辛 | 短尾细辛Asarum caudigerellum C.Y.Cheng et C.S.Yang |
|  |  | 尾花细辛Asarum caudigerum Hance |
|  |  | 青城细辛Asarum splendens(Maekawa) C.Y.Cheng et  C.S.Yang |
|  |  | 双叶细辛Asarum caulescens Maxim |
| 16 | 乌金七 | 双叶细辛Asarum caulescens Maxim. |
| 17 | 杜衡 | 杜衡Asarum forbesii Maxim. |
|  |  | 宜昌细辛Asarum ichangense C.Y.Cheng et C.S.Yang |
| 18 | 湘细辛 | 杜衡Asarum forbesii Maxim |
|  |  | 小叶马蹄香Asarum ichangense C.Y.Cheng&C.S.Yang |
|  |  | 五岭细辛Asarum wulingense C.F.Liang |
| 19 | 细辛 | 北细辛Asarum heterotropoides Fr.Schmidt var.mandshu ricum(Maxim.)Kitag. |
|  |  | 汉城细辛Asarum sieboldii Miq.var.seoulense Nakai |
|  |  | 华细辛Asarum sieboldii Miq |
| 20 | 甘肃细辛 | 单叶细辛Asarun himalacum Hook.f.et Thoms. |
| 21 | 南坪细辛 | 单叶细辛Asarum himalaicum Hook.f.et Thoms.ex Klotzsch |
| 22 | 毛细辛 | 单叶细辛Asarum himalaicum Hook.f.et Thoms.ex Klotzsch |
| 23 | 金耳环 | 金耳环Asarum insigne Diels |
| 24 | 山慈菇 | 山慈菇Asarum sagittarioides C.F.Liang |

**Appendix-2**

**List of Chinese patent medicines containing *Asarum* genus**

| 序号 | 药品名称 | 含马兜铃科药材 |
| --- | --- | --- |
| 1 | 八宝镇惊丸 | 细辛 |
| 2 | 百草油 | 细辛 |
| 3 | 保安万灵丹(丸) | 细辛 |
| 4 | 鼻炎灵片 | 细辛 |
| 5 | 鼻炎灵丸 | 细辛 |
| 6 | 鼻炎片 | 细辛 |
| 7 | 鼻渊舒胶囊 | 细辛 |
| 8 | 鼻渊舒口服液 | 细辛 |
| 9 | 参贝咳喘丸 | 细辛 |
| 10 | 参芪颗粒 | 细辛 |
| 11 | 参茸木瓜药酒 | 细辛 |
| 12 | 参茸全虫酒 | 细辛 |
| 13 | 参三七伤药片 | 细辛 |
| 14 | 参麝活络丸 | 细辛 |
| 15 | 参仙升脉口服液 | 细辛 |
| 16 | 柴辛感冒注射液 | 细辛 |
| 17 | 产灵丸(产灵丹) | 细辛 |
| 18 | 沉香安神胶囊 | 细辛 |
| 19 | 沉香安神散 | 细辛 |
| 20 | 沉香散 | 细辛 |
| 21 | 齿痛消炎灵颗粒 | 细辛 |
| 22 | 川芎茶调冲剂 | 细辛 |
| 23 | 川芎茶调袋泡剂 | 细辛 |
| 24 | 川芎茶调颗粒 | 细辛 |

| 序号 | 药品名称 | **含马兜铃科药材** |
| --- | --- | --- |
| 25 | 川芎茶调口服液 | 细辛 |
| 26 | 川芎茶调片 | 细辛 |
| 27 | 川芎茶调散 | 细辛 |
| 28 | 川芎茶调丸 | 细辛 |
| 29 | 川芎茶调丸(浓缩丸) | 细辛 |
| 30 | 川芎清脑颗粒 | 细辛 |
| 31 | 大活络胶囊 | 细辛 |
| 32 | 大活络丸 | 细辛 |
| 33 | 丹葛颈舒胶囊 | 细辛 |
| 34 | 丹桂香颗粒 | 细辛 |
| 35 | 丹珍头痛胶囊 | 细辛 |
| 36 | 跌打风湿酒 | 细辛 |
| 37 | 丁细牙痛胶囊 | 细辛 |
| 38 | 定坤丹 | 细辛 |
| 39 | 冬菀止咳颗粒 | 细辛 |
| 40 | 独活寄生合剂 | 细辛 |
| 41 | 独活寄生颗粒 | 细辛 |
| 42 | 独活寄生丸 | 细辛 |
| 43 | 儿童清肺丸 | 细辛 |
| 44 | 二益丸 | 细辛 |
| 45 | 发汗解热丸 | 细辛 |
| 46 | 风湿安泰片 | 细辛 |
| 47 | 风湿酒 | 细辛 |
| 48 | 风痛丸 | 细辛 |
| 49 | 佛山人参再造丸 | 细辛 |
| 50 | 复方半夏片 | 细辛 |
| 51 | 复方杜仲壮腰胶囊 | 细辛 |
| 52 | 复方感冒胶囊 | 细辛 |
| 53 | 复方感冒片 | 细辛 |
| 54 | 复方罗汉果含片 | 细辛 |

| **序号** | **药品名称** | **含马兜铃科药材** |
| --- | --- | --- |
| 55 | 感特灵胶囊 | 细辛 |
| 56 | 感特灵片 | 细辛 |
| 57 | 古威活络酊 | 细辛 |
| 58 | 固肾补气散 | 细辛 |
| 59 | 寒喘丸 | 细辛 |
| 60 | 寒湿痹颗粒 | 细辛 |
| 61 | 寒湿痹片 | 细辛 |
| 62 | 喉炎丸 | 细辛 |
| 63 | 猴枣牛黄散 | 细辛 |
| 64 | 回天再造丸 | 细辛 |
| 65 | 活络丸 | 细辛 |
| 66 | 活血镇痛胶囊 | 细辛 |
| 67 | 寄生追风酒(寄生追风液) | 细辛 |
| 68 | 解表追风丸 | 细辛 |
| 69 | 解毒利咽丸 | 细辛 |
| 70 | 解暑片 | 细辛 |
| 71 | 金关片 | 细辛 |
| 72 | 筋骨跌打丸 | 细辛 |
| 73 | 筋骨康片 | 细辛 |
| 74 | 惊风丸 | 细辛 |
| 75 | 九龙化风丸 | 细辛 |
| 76 | 九味羌活颗粒 | 细辛 |
| 77 | 九味羌活口服液 | 细辛 |
| 78 | 九味羌活片 | 细辛 |
| 79 | 九味羌活丸 | 细辛 |
| 80 | 救急行军胶囊 | 细辛 |
| 81 | 救急行军散 | 细辛 |
| 82 | 抗栓再造丸 | 细辛 |
| 83 | 克痢痧胶囊 | 细辛 |
| 84 | 利鼻片 | 细辛 |

| 序号 | 药品名称 | **含马兜铃科药材** |
| --- | --- | --- |
| 85 | 六经头痛片 | 细辛 |
| 86 | 龙灯胶囊 | 细辛 |
| 87 | 鸬鹚涎丸 | 细辛 |
| 88 | 鹭鸶咯丸 | 细辛 |
| 89 | 罗补甫克比日丸 | 细辛 |
| 90 | 麻黄止嗽丸 | 细辛 |
| 91 | 明目羊肝丸 | 细辛 |
| 92 | 辟瘟片 | 细辛 |
| 93 | 平肝舒络丸 | 细辛 |
| 94 | 七味解痛口服液 | 细辛 |
| 95 | 芪丹通络颗粒 | 细辛 |
| 96 | 庆余辟瘟丹 | 细辛 |
| 97 | 驱风苏合丸 | 细辛 |
| 98 | 泉州百草曲 | 细辛 |
| 99 | 人参再造丸 | 细辛 |
| 100 | 人参再造丸(蜜丸) | 细辛 |
| 101 | 人参再造丸(浓缩丸 | 细辛 |
| 102 | 三鞭温阳胶囊 | 细辛 |
| 103 | 三余神曲 | 细辛 |
| 104 | 散风活络丸 | 细辛 |
| 105 | 散风活络丸(浓缩丸) | 细辛 |
| 106 | 痧气丸 | 细辛 |
| 107 | 伤痛宁片 | 细辛 |
| 108 | 蛇咬丸 | 细辛 |
| 109 | 十一味参芪片 | 细辛 |
| 110 | 石辛含片 | 细辛 |
| 111 | 食道平散 | 细辛 |
| 112 | 疏风再造丸 | 细辛 |
| 113 | 署症片 | 细辛 |
| 114 | 双辛鼻窦炎颗粒 | 细辛 |

| **序号** | **药品名称** | **含马兜铃科药材** |
| --- | --- | --- |
| 115 | 痰喘半夏颗粒 | 细辛 |
| 116 | 天草颗粒 | 细辛 |
| 117 | 天麻壮骨丸 | 细辛 |
| 118 | 调元大补二十五味汤散 | 细辛 |
| 119 | 跳骨片 | 细辛 |
| 120 | 通络活血丸 | 细辛 |
| 121 | 通天口服液 | 细辛 |
| 122 | 同仁大活络丸 | 细辛 |
| 123 | 透骨镇风丸(透骨镇风丹) | 细辛 |
| 124 | 万灵片 | 细辛 |
| 125 | 万通筋骨片 | 细辛 |
| 126 | 胃病丸(胃病丹) | 细辛 |
| 127 | 胃可安胶囊 | 细辛 |
| 128 | 温中止泻丸 | 细辛 |
| 129 | 乌梅丸 | 细辛 |
| 130 | 无敌丹胶囊 | 细辛 |
| 131 | 无敌药酒 | 细辛 |
| 132 | 熄风通络头痛片 | 细辛 |
| 133 | 消肿止痛酊 | 细辛 |
| 134 | 小儿保安丸 | 细辛 |
| 135 | 小儿肺闭宁片 | 细辛 |
| 136 | 小儿咳喘颗粒 | 细辛 |
| 137 | 小儿治哮灵片 | 细辛 |
| 138 | 小青龙合剂(口服液) | 细辛 |
| 139 | 小青龙胶囊 | 细辛 |
| 140 | 小青龙颗粒 | 细辛 |
| 141 | 心脑联通胶囊 | 细辛 |
| 142 | 心无忧片 | 细辛 |
| 143 | 辛芳鼻炎胶囊 | 细辛 |
| 144 | 辛芩颗粒 | 细辛 |

| 序号 | 药品名称 | **含马兜铃科药材** |
| --- | --- | --- |
| 145 | 辛芩片 | 细辛 |
| 146 | 醒脑再造胶囊 | 细辛 |
| 147 | 醒脑再造丸 | 细辛 |
| 148 | 牙痛宁滴丸 | 细辛 |
| 149 | 牙痛清火口服液 | 细辛 |
| 150 | 羊痫疯癫丸 | 细辛 |
| 151 | 养血清脑颗粒 | 细辛 |
| 152 | 养血清脑丸 | 细辛 |
| 153 | 药酒丸 | 细辛 |
| 154 | 伊痛舒合剂 | 细辛 |
| 155 | 伊痛舒注射液 | 细辛 |
| 156 | 薏辛除湿止痛胶囊 | 细辛 |
| 157 | 银盏心脉滴内 | 细辛 |
| 158 | 云香祛风止痛酊(云香精) | 细辛 |
| 159 | 再造丸 | 细辛 |
| 160 | 镇脑宁胶囊 | 细辛 |
| 161 | 正天胶囊 | 细辛 |
| 162 | 正天丸 | 细辛 |
| 163 | 止咳化痰丸 | 细辛 |
| 164 | 止痛水 | 细辛 |
| 165 | 中风再造丸 | 细辛 |
| 166 | 珠贝定喘丸 | 细辛 |
| 167 | 状元红药酒 | 细辛 |
| 168 | 追风透骨片 | 细辛 |
| 169 | 追风透骨丸 | 细辛 |
| 170 | 紫灯胶囊 | 细辛 |
| 171 | 散风通窍滴丸 | 细辛 |
| 172 | 辛芩胶囊 | 细辛 |
| 173 | 寒湿痹胶囊 | 细辛 |
| 174 | 九味羌活软胶囊 | 细辛 |

| 序号 | 药品名称 | 含马兜铃科药材 |
| --- | --- | --- |
| 175 | 丹玉通脉颗粒 | 细辛 |
| 176 | 清脑止痛胶囊 | 细辛 |

备注：以上不包括外用制剂。

**Appendix-3**

**List of Chinese patent medicines containing *Aristolochia* genus**

| 序号 | 药品名称 | **含马兜铃属药材** |
| --- | --- | --- |
| 1 | 通迪胶囊 | 大青木香 |
| 2 | 复方胃痛胶囊 | 九月生 |
| 3 | 喘息灵胶囊 | 马兜铃 |
| 4 | 二十味疏肝胶囊 | 马兜铃 |
| 5 | 肺安片 | 马兜铃 |
| 6 | 复方蛇胆川贝散 | 马兜铃 |
| 7 | 鸡鸣丸 | 马兜铃 |
| 8 | 鸡苏丸 | 马兜铃 |
| 9 | 七十味松石丸 | 马兜铃 |
| 10 | 青果止嗽丸 | 马兜铃 |
| 11 | 润肺化痰丸(鸡鸣丸) | 马兜铃 |
| 12 | 十三味疏肝胶囊 | 马兜铃 |
| 13 | 十四味疏肝胶囊 | 马兜铃 |
| 14 | 四十二味疏肝胶囊 | 马兜铃 |
| 15 | 胃福颗粒 | 马兜铃 |
| 16 | 小儿热咳口服液 | 马兜铃 |
| 17 | 止嗽化痰丸 | 马兜铃 |
| 18 | 京制咳嗽痰喘丸 | 马兜铃 |
| 19 | 消咳平喘口服液 | 马兜铃 |
| 20 | 止嗽化痰胶囊 | 蜜马兜铃 |
| 21 | 二十九味能消散 | 木香马兜铃 |
| 22 | 二十五味绿绒蒿胶囊 | 木香马兜铃 |
| 23 | 二十五味绿绒蒿丸 | 木香马兜铃 |
| 24 | 二十五味松石丸 | 木香马兜铃 |

| **序号** | **药品名称** | **含马兜铃属药材** |
| --- | --- | --- |
| 25 | 二十五味余甘子丸 | 木香马兜铃 |
| 26 | 二十五味竺黄散 | 木香马兜铃 |
| 27 | 风湿塞隆胶囊 | 木香马兜铃 |
| 28 | 风湿止痛丸 | 木香马兜铃 |
| 29 | 肝畅胶囊 | 木香马兜铃 |
| 30 | 九味牛黄丸 | 木香马兜铃 |
| 31 | 七味红花殊胜散 | 木香马兜铃 |
| 32 | 七味红花殊胜丸 | 木香马兜铃 |
| 33 | 清肺止咳丸 | 木香马兜铃 |
| 34 | 四味止泻木汤散 | 木香马兜铃 |
| 35 | 五味渣驯丸 | 木香马兜铃 |
| 36 | 和胃降逆胶囊 | 天仙藤 |
| 37 | 复方风湿药酒 | 寻骨风 |
| 38 | 复方拳参片 | 寻骨风 |
| 39 | 祛风除湿药酒 | 寻骨风 |
| 40 | 三蛇药酒 | 寻骨风 |
| 41 | 神农药酒 | 寻骨风 |
| 42 | 益肾蠲痹丸 | 寻骨风 |
| 43 | 杜仲壮骨胶囊 | 寻骨风 |
| 44 | 杜仲壮骨丸 | 寻骨风 |
| 45 | 保胃胶囊 | 朱砂莲 |
| 46 | 金朱止泻片 | 朱砂莲 |
| 47 | 朱砂莲胶囊 | 朱砂莲 |

备注：以上不包括外用制剂。
